# Innate And Adaptive Immunity are Progressively Activated in Parallel with Renal Injury in the 5/6 Renal Ablation Model

**DOI:** 10.1038/s41598-017-02915-6

**Published:** 2017-06-09

**Authors:** Camilla Fanelli, Simone C. A. Arias, Flavia G. Machado, Jessica K. Okuma, Denise M. A. C. Malheiros, Hatylas Azevedo, Carlos A. Moreira-Filho, Niels O. S. Camara, Clarice K. Fujihara, Roberto Zatz

**Affiliations:** Faculty of Medicine, University of São Paulo, São Paulo, Brazil

## Abstract

The mechanisms triggering renal inflammation in chronic kidney disease (CKD) are unclear. We performed a detailed analysis of the time course of innate and adaptive immunity activation in the 5/6 renal ablation (Nx) model. Munich-Wistar rats undergoing Nx were studied 15, 60 and 120 days after ablation. Hypertension, albuminuria, creatinine retention, interstitial expansion and infiltration by macrophages and T-lymphocytes were already evident 15 days after Nx. PCR-array was used to screen for altered gene expression, whereas gene and protein expressions of TLR4, CASP1, IL-1β and NLRP3 were individually assessed. *Tlr4*, *Tlr5*, *Lbp*, *Nlrp3*, *Casp1*, *Irf7* and *Il1b* were already upregulated 15 days after Nx, while activation of *Tlr2*, *Tlr7*, *Tlr9*, *Nod2*, *Tnf* and *Il6* was seen after 60 days post-ablation. The number of genes related to innate or adaptive immunity grew steadily with time. These observations indicate that parallel activation of innate and adaptive immunity antecedes glomerular injury and involves a growing number of intricate signaling pathways, helping to explain the difficulty in detaining renal injury in Nx as CKD advances, and, stressing the need for early treatment. Additionally, these findings may contribute to the search of therapeutic targets specific for advanced phases of CKD.

## Introduction

Inordinate production of cytokines, chemokines and growth factors, renal infiltration by macrophages (Mϕ), T-lymphocytes (T-Ly), fibroblasts and myofibroblasts, as well as renal interstitial fibrosis have been described in chronic kidney disease (CKD), regardless of etiology^1–4^. Accordingly, treatment with anti-inflammatory drugs, such as mycophenolate mofetil (MMF), and even nonsteroidal anti-inflammatory drugs, was shown to be renoprotective in widely employed models of CKD such as 5/6 nephrectomy (Nx), chronic nitric oxide inhibition, and streptozotocin-induced diabetes^5–9^. However, the reasons why inflammation develops in the renal tissue even when CKD is not initiated by a clear cut immune dysfunction or by the presence of foreign antigens are presently unclear.

Inflammation is inextricably related with both the innate and adaptive branches of the immune response. Innate immunity refers to quick and nonspecific defense mechanisms involving phagocytes such as Mϕ and neutrophils, evolutionarily conserved “pattern recognition receptors” and intracellular pathways that respond to pathogen-associated molecular patterns (PAMPs), such as bacterial flagellin and lipopolysaccharides (LPSs), and damage-associated molecular patterns (DAMPs), such as membrane debris and cytosolic double-stranded DNA. Adaptive immunity is a slower and more complex set of mechanisms that comprise antigen recognition, processing and, ultimately, elimination by specialized B-Ly (antibody producers) and T-Ly (cytotoxic)^10–13^.

Activation of innate immunity pathways in CKD may represent an important link between nonspecific insults, for instance, glomerular wall stretching or tubular exposure to high protein concentrations, and the late development of glomerulosclerosis (GS) and renal fibrosis^11–15^. Several pathways related to innate immune response were shown to be activated in the progression of CKD, such as the NF-κB system, the Toll-like receptor (TLRs 1–9) signaling pathway, the NOD2 receptor and the NLRP3 inflammasome^11, 12, 16–26^. The latter is required for the conversion of pro-caspase-1 into its active form, caspase-1 (CASP1), and the resulting release of proinflammatory interleukins (IL-1β and IL-18)^16, 23, 25, 26^. NLRP3 inflammasome and IL-1β are overexpressed shortly after renal ablation and may be crucial to the establishment of renal injury in the Nx model^22^. We and others showed evidence that the NF-κB system is activated in Nx, and that its chronic inhibition exerts a renoprotective effect in CKD progression^18, 20, 21, 27^. Additional studies indicated that *Tlr2* and *Tlr4* knockout (KO) mice develop less tubulointerstitial nephritis and renal fibrosis^17^.

Whatever the nature of the initial insult, the resulting cell damage may further stimulate innate immunity, leading to a positive feedback that may promote engagement of adaptive immunity, perpetuating inflammation and culminating in the establishment of end-stage renal disease (ESRD). If this hypothesis is correct, progressive activation of innate immunity pathways should parallel or even precede the development of renal injury in experimental models of CKD.

Timing may be crucial to the efficacy of CKD treatment. Early inhibition of adaptive immunity with MMF, an antilymphocyte agent, was shown to prevent renal injury in the Nx model^7, 28^. However, MMF treatment had no effect when started a month after 5/6 ablation^29^. Similar findings were obtained with the AT1 receptor blocker, losartan^29, 30^. The reason why these treatments lose their efficacy when initiated late in the course of renal disease is currently unknown. One possible explanation is that progressive recruitment of a growing number of powerful, often redundant inflammatory pathways, involving angiotensin II, as well as innate and adaptive immunity, among other systems, may render it extremely difficult to arrest kidney injury with pharmacologic interventions, even when two or more agents are combined.

In the present study, we examined the time course of a broad range of genes related to both innate and adaptive immune responses, from 15 to 120 days after 5/6 renal ablation, correlating their expression with the extent of renal damage. In this manner, we were able to show that concerted activation of innate and adaptive immunity starts very early in the course of the Nx model, gaining strength and complexity with time. In addition, and with the help of bioinformatic analysis, we propose a scheme that may allow us to focus on more specific pathways and time points in the struggle to arrest CKD even at advanced phases.

## Results

### General Parameters

Results for body weight (BW), tail-cuff pressure (TCP), albuminuria (U_alb_V) and serum creatinine concentration (S_Cr_) are presented in Table 1. Consistent with previous studies^1, 31, 32^ body growth gain was slower in nephrectomized animals when compared to Sham. Nx animals also exhibited a progressive and significant elevation of TCP, U_alb_V and S_Cr_, compared to Sham.

**Table 1:** Body weight, blood pressure and renal function 15, 60 and 120 days after Nx.

Systemic/Renal Parameters	Sham	Nx 15d	Nx 60d	Nx 120d
**BW** (g)	396 ± 9	225 ± 9^a^	268 ± 5^ab^	304 ± 6^abc^
**TCP** (mmHg)	142 ± 1	188 ± 5^a^	209 ± 3^ab^	217 ± 4^ab^
**U**_**alb**_ **V** (mg/24 h)	8 ± 2	108 ± 15^a^	112 ± 18^a^	177 ± 13^ab^
**S**_**cr**_ (mg/dL)	0.62 ± 0.03	0.83 ± 0.06^a^	1.09 ± 0.06^ab^	1.23 ± 0.07^ab^

Results presented as Mean ± SE. Differences among groups were analyzed by one-way ANOVA with Newman-Keuls post test. ^**a**^p < 0.05 vs. Sham, ^**b**^p < 0.05 vs. Nx 15d, ^**c**^p < 0.05 vs. Nx 60d.

**Figure 1:**
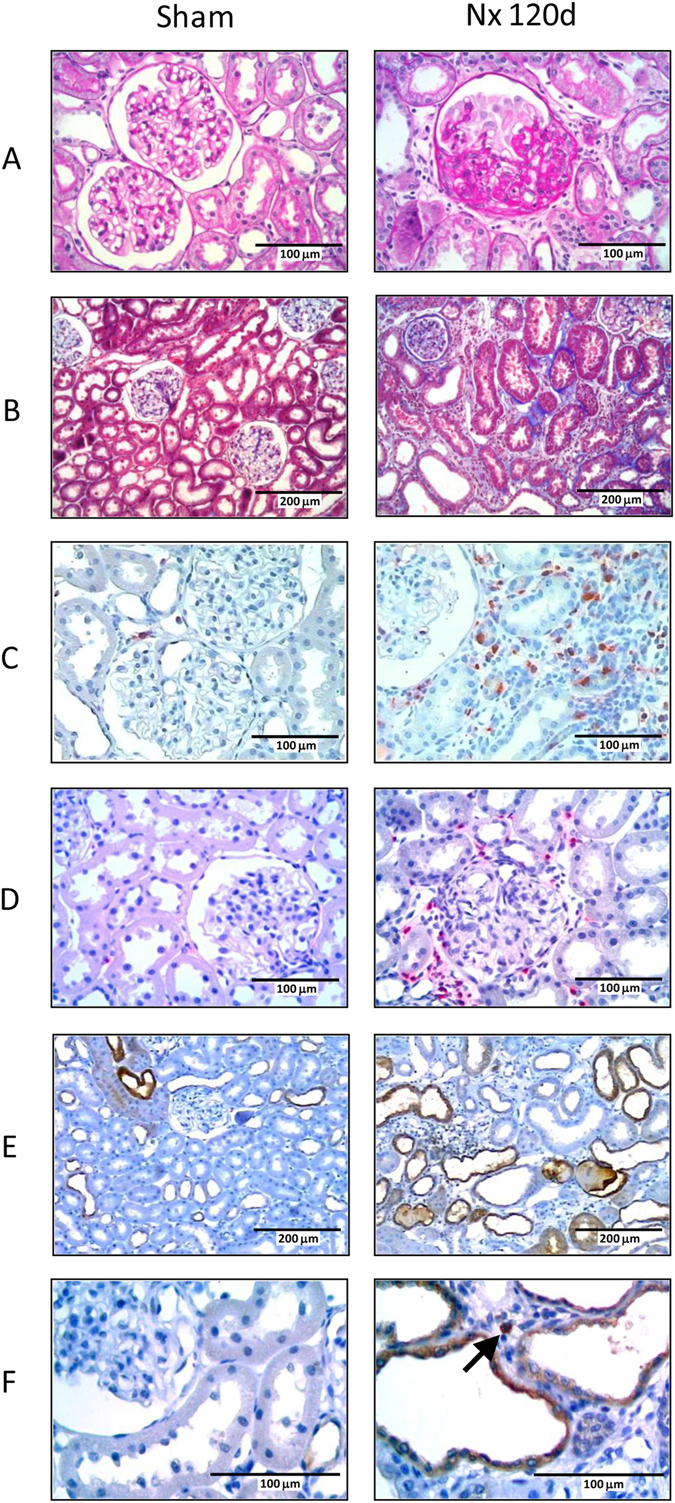
Illustrative microphotographs of renal tissue from Sham (left) and Nx (right) rats, 120 days after renal ablation. PAS staining (**A**) was used to analyze the glomerular architecture, thus identifying the presence and intensity of glomerulosclerosis. Masson’s trichrome (**B**) was employed to evaluate the percentage of renal cortical interstitial area. Immunohistochemistry was performed to identify and quantify the renal interstitial infiltration of macrophages (**C**) and T-Lymphocytes (**D**), both stained in red, as well as the percentage of TLR4^+^ renal tubules (**D**) and the presence of TLR4^+^ interstitial cells (**E**), both stained in dark-brown.

**Figure 2:**
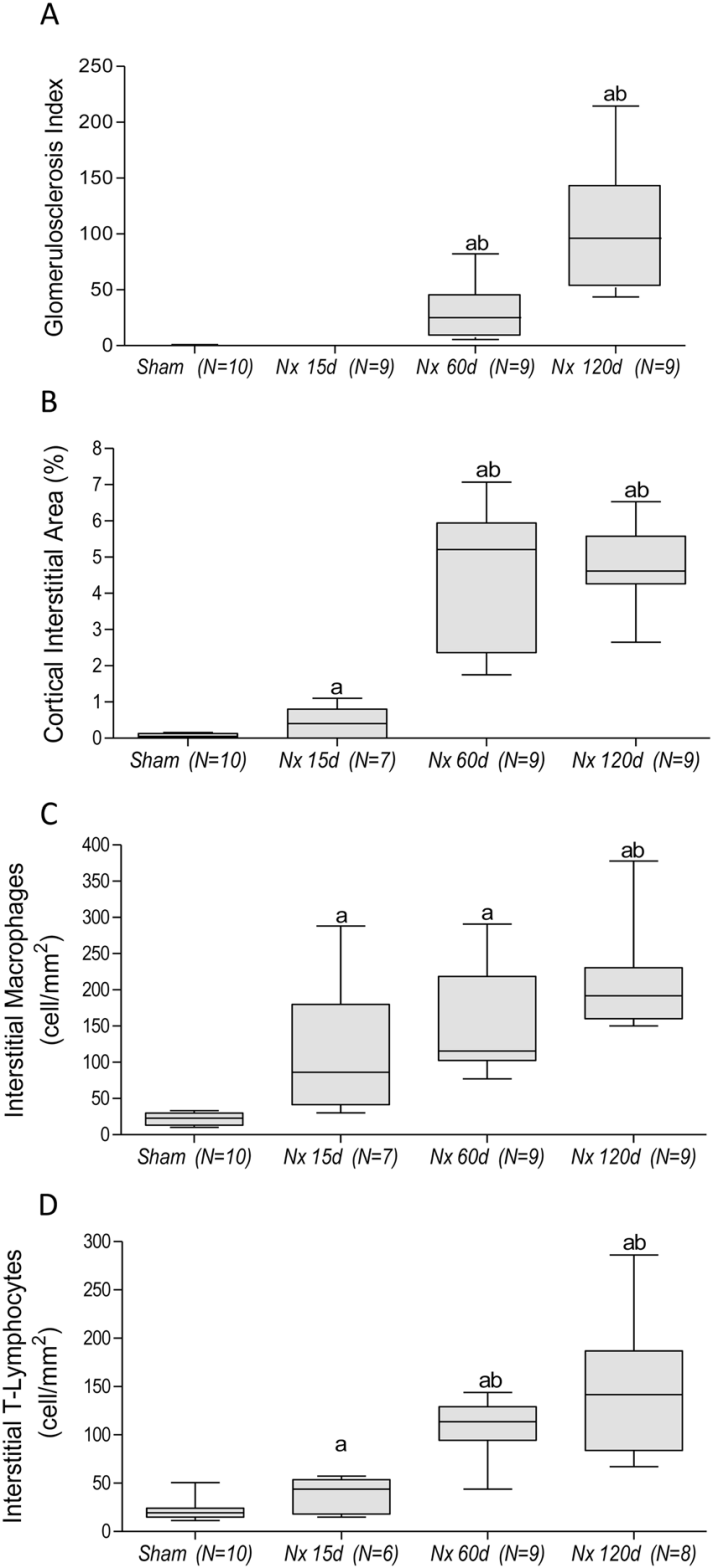
Quantitative analysis of (**A**) Glomerulosclerosis Index, (**B**) Percentage of cortical interstitial area, and the extent of renal cortical interstitial infiltration by Macrophages (**C**) and T-Lymphocytes (**D**). Results are presented as median plus range. The number of analyzed animals is represented next to the name of each group. Differences among groups were assessed by one-way ANOVA with Newman-Keuls post test. ^a^p < 0.05 vs. Sham, ^b^p < 0.05 vs. Nx 15d, ^c^p < 0.05 vs. Nx 60d.

No glomerulosclerosis (GS) was observed in Nx 15 days after renal ablation. However, 60 days after Nx, the GS index (GSI) was significantly higher than in Sham, exhibiting further increase 120 days after surgery (Figs 1A and 2A). Likewise, a significant increase of the fractional cortical interstitial area (INT) was seen in Nx after 15 days of Nx, with progressive increase along the rest of the study (Figs 1B and 2B).

The intensity of interstitial Mϕ infiltration was significantly higher in Nx than in Sham rats 15 days after nephrectomy, increasing progressively throughout the study (Figs 1C and 2C). The profile of renal interstitial infiltration by T-Ly followed that observed for Mϕ (Figs 1D and 2D).

### Innate and adaptive immune responses

**Figure 3:**
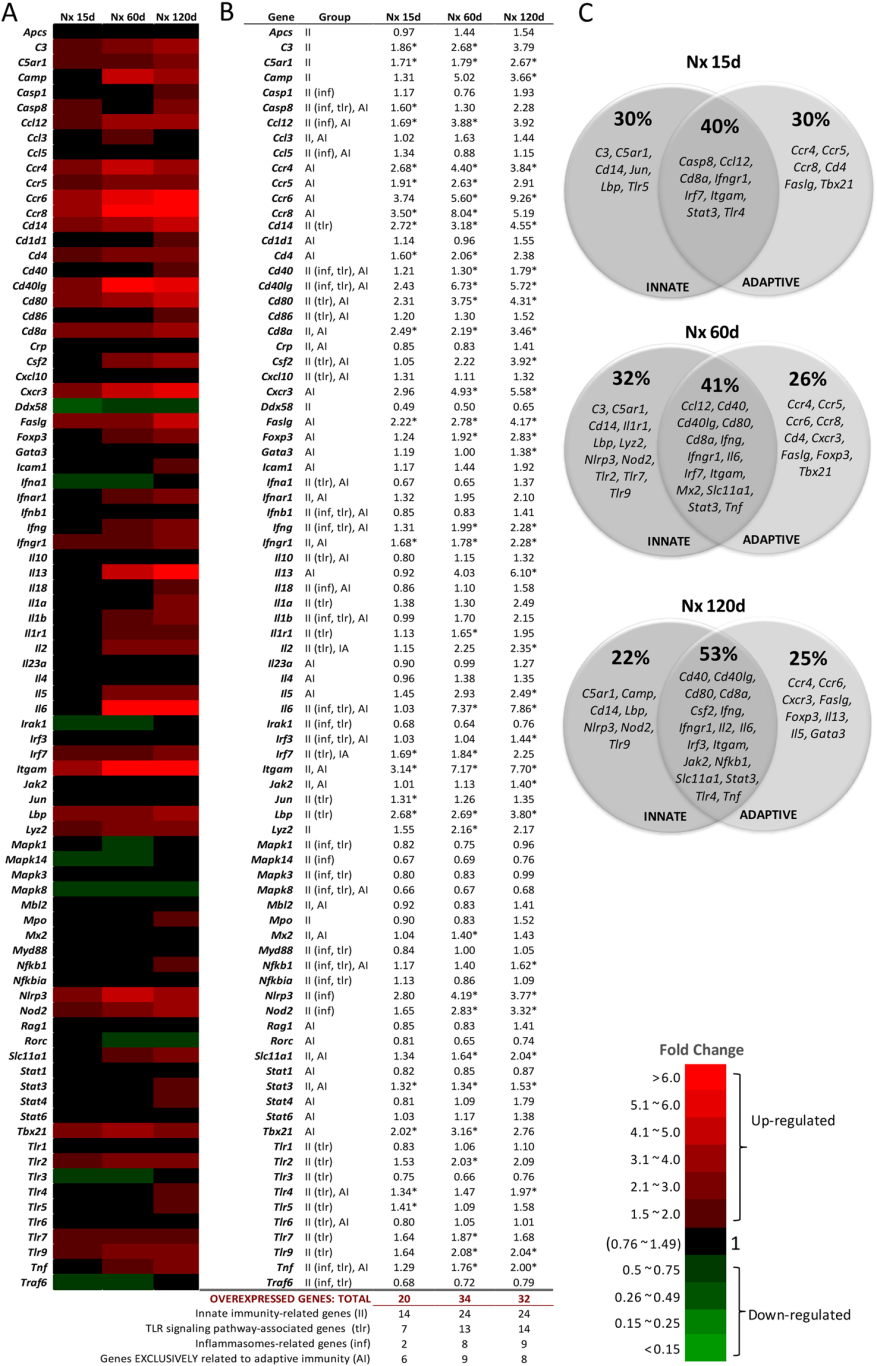
Heat map representation of PCR-array analysis (**A**) with the respective fold change value (mean) of each studied gene (**B**). Venn diagrams (**C**) illustrate the relative participation of overexpressed genes related to innate and adaptive immune responses at each time point. For these experiments, cDNA samples were obtained from nine different animals per group: Sham, Nx 15d, Nx 60d and Nx 120d. These cDNA was than distributed among three pools per group, containing genetic material of three different animals each. For statistical analysis we considered N = 3. Differences between Nx and Sham were assessed by Student’s t-test (*p < 0.05 vs. Sham), using the Rt2 Profiler PCR Array Analysis software, Version 3.5™ (Qiagen).

A heat map representation of 84 genes related to innate and adaptive immunity, assessed by PCR-array analysis, is shown in Fig. 3A, and quantitatively expressed in Fig. 3B. Fifteen days after ablation, the kidneys of Nx rats exhibited 20 significantly up-regulated genes associated to inflammation, 6 of which were exclusively related to the adaptive immune response, and 14 were related to the innate immune response, exclusively or not. Of these, 7 were part of the TLR, and 2 belonged to the NOD2 and/or NLRP3 inflammasome, signalling pathways. Sixty days after renal ablation, we found 34 up-regulated genes, of which 9 were exclusively related to adaptive immunity, and 24 were related to the innate immune response, exclusively or not. Of the latter, 13 were associated with the TLR signaling pathway and 8 with the NOD2 and/or NLRP3 inflammasomes systems. Finally, 120 days after nephrectomy, we observed a total of 32 overexpressed genes. Eight of these were exclusively related to adaptive immunity, and 24 were related to the innate immune response, exclusively or not. Of these, 14 were associated with the TLRs and 9 to the inflammasomes signaling pathways. Venn diagrams (Fig. 3C) illustrate the relative participation of overexpressed genes related to innate and adaptive immune responses at each time point.

**Figure 4:**
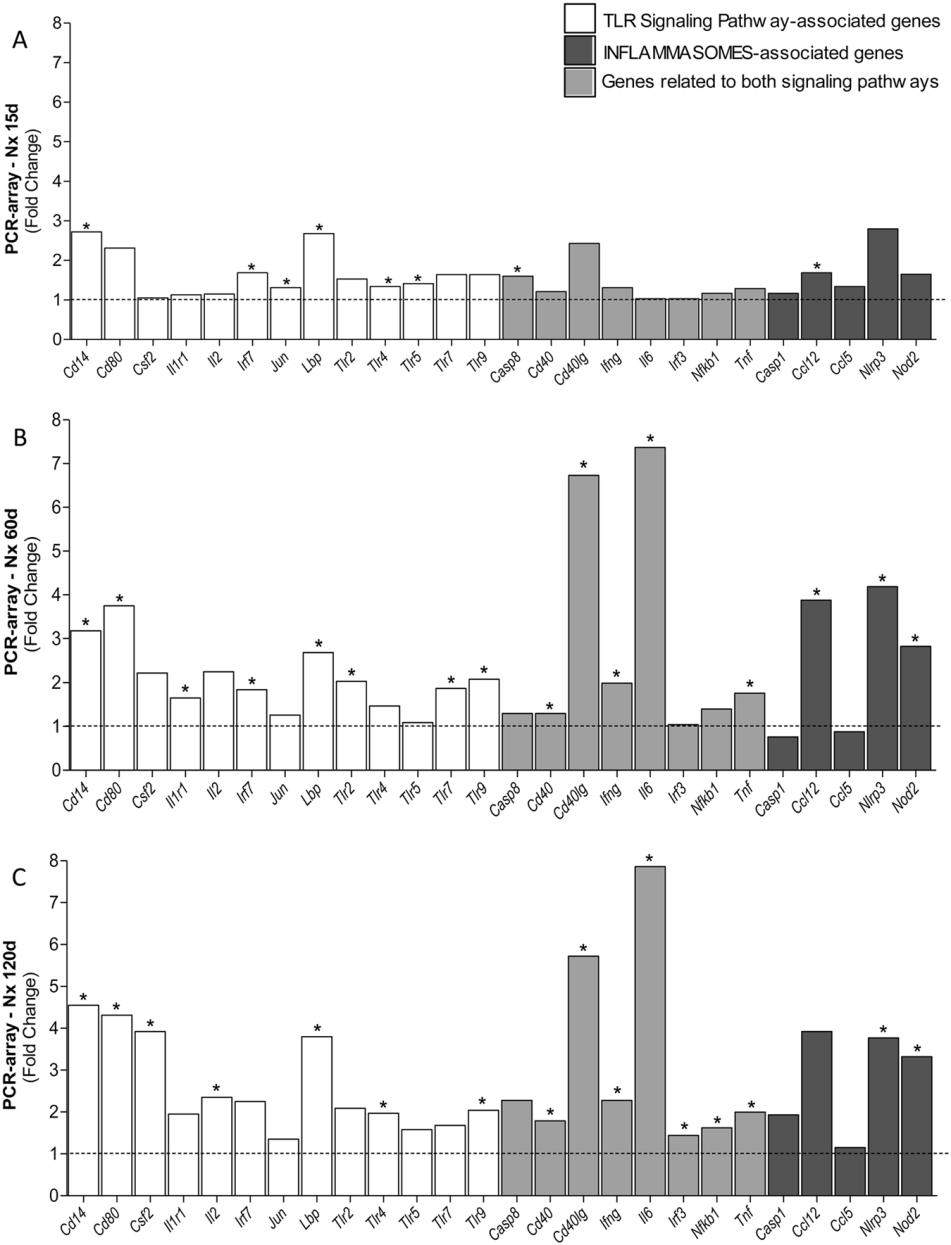
Bar graph representation of the PCR-array analysis of genes related to the TLR and inflammasome pathways throughout the study. For these experiments, cDNA samples were obtained from nine different animals per group: Sham, Nx 15d, Nx 60d and Nx 120d. These cDNA was than distributed among three pools per group, containing genetic material of three different animals each. For statistical analysis we considered N = 3. Results are presented as mean of fold change, compared to Sham animals. Differences between Nx and Sham were assessed by Student’s t-test (*p < 0.05 vs. Sham), using the Rt2 Profiler PCR Array Analysis software, Version 3.5™ (Qiagen).

**Figure 5:**
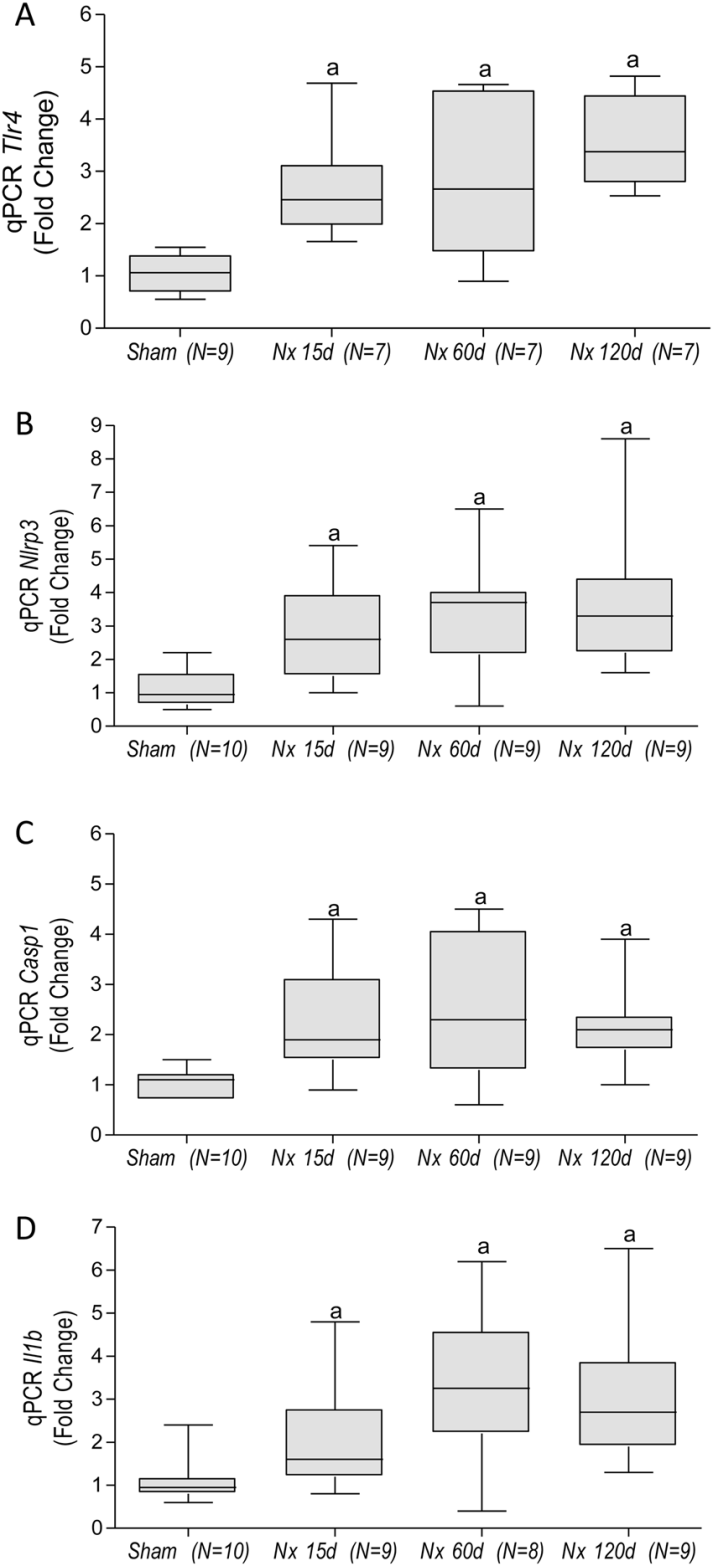
Quantitative PCR analysis (qPCR) for *Tlr4* (**A**), *Nlrp3* (**B**), *Casp1* (**C**) and *Il1b* (**D**) gene expression. Results are presented as median plus range. The number of analyzed animals is represented next to the name of each group. Differences among groups were analyzed by one-way ANOVA with Newman-Keuls post test. ^a^p < 0.05 vs. Sham, ^b^p < 0.05 vs. Nx 15d, ^c^p < 0.05 vs. Nx 60d.

**Figure 6:**
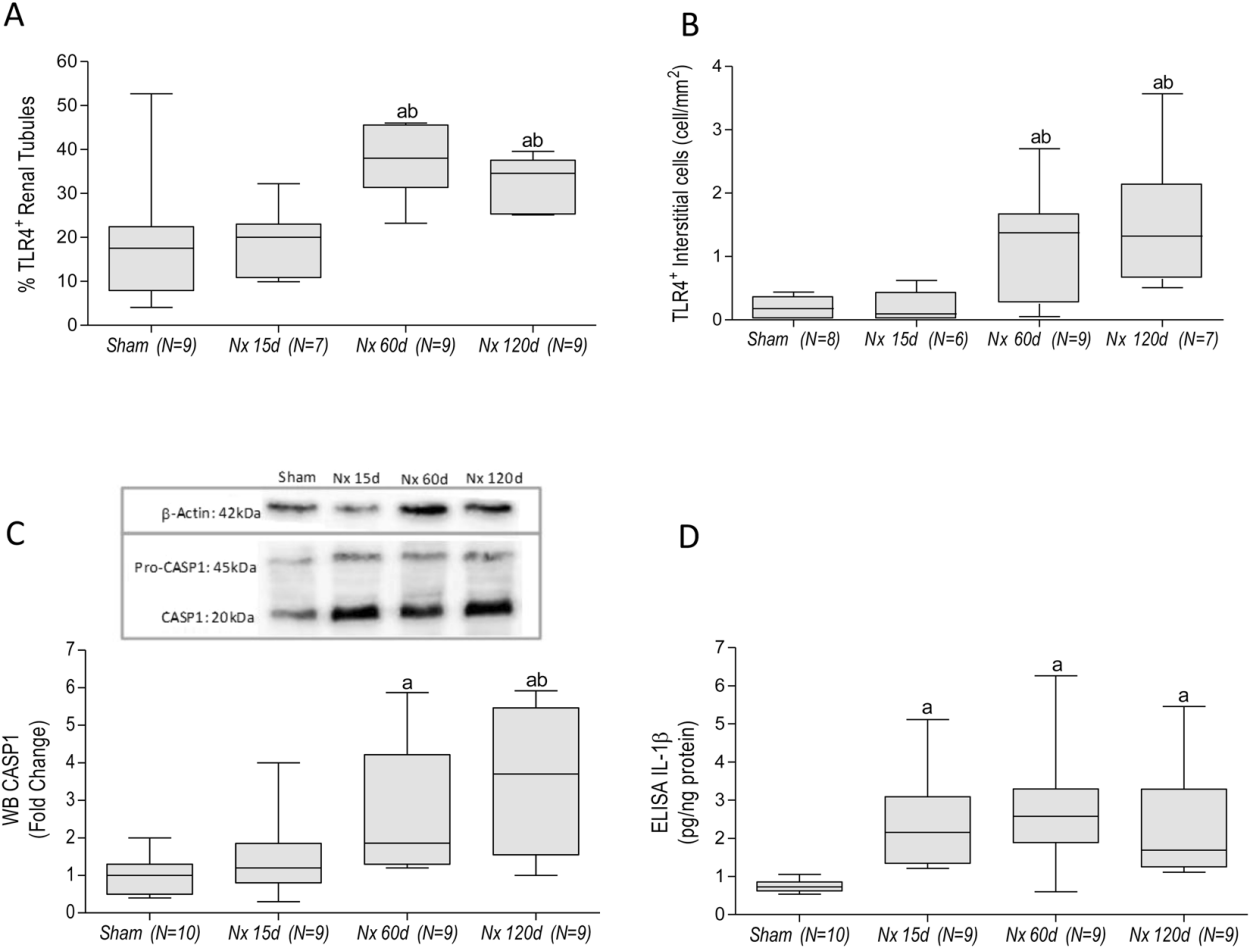
Quantitative analysis of immunohistochemistry (IHQ) for TLR4 in the renal tubular (**A**) and in the renal interstitial (**B**) compartments. Representative Western Blot (WB) and its relative band intensity quantification (**C**). Quantitative analysis of ELISA for IL-1β (**D**). Results are presented as median plus range. The number of analyzed animals is represented next to the name of each group. Differences among groups were analyzed by one-way ANOVA with Newman-Keuls post test. ^a^p < 0.05 vs. Sham, ^b^p < 0.05 vs. Nx 15d, ^c^p < 0.05 vs. Nx 60d. The complete blot for Casp-1 is provided under “Supplementary Material”.

Figure 4 shows the expression of genes related to TLR, inflammasomes or both pathways, at each time of the study. Both the number of overexpressed genes and the intensity (fold change) increased continuously with time. The overexpression of *Tlr4*, *Nlrp3*, *Casp1* and *Il1b* genes was confirmed by individual qPCR (Fig. 5). Accordingly, increased amounts of TLR4 protein were detected by immunohistochemistry (IHC) in both renal tubular (Figs 1E and 6A) and interstitial cells (Figs 1F and 6B) after 60 and 120 days after renal ablation. The renal abundance of Caspase-1 (CASP1) followed a similar pattern (Fig. 6C), whereas that of IL-1β was increased at 15, 60 and 120 days after ablation (Fig. 6D).

**Figure 7:**
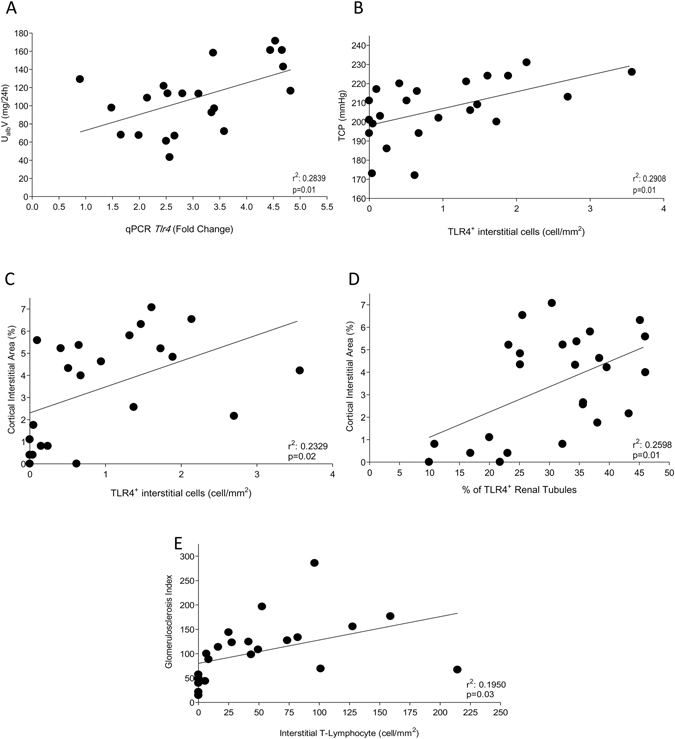
Scatterplot graphs were used to represent the correlations between: (**A**) Albuminuria (U_alb_V,) and the*Tlr4* gene expression, (**B**) Tail-Cuff Pressure (TCP) and the number of TLR4^+^ interstitial cells, Percentage of cortical interstitial area and both (**C**) the number of TLR4^+^ interstitial cells and (**D**) the percentage of TLR4^+^ tubules, and finally, (**E**) the Gomerulosclerosis Index (GS) and interstitial T-Lymphocyte infiltration, in 25 Nx rats. Significant correlations were established according to the Pearson’s coefficient, when p < 0.05.

Figuer 7 depicts positive linear correlation between *Tlr4* gene expression and albumin excretion rate (A), interstitial TLR4^+^ cells and either TCP (B) and percent interstitial area (C), as well as percent of TLR4^+^ tubules and percent interstitial area (D). A positive linear correlation was also observed between the density of T-Ly infiltration and the GS index (Fig. 7E).

### Macrophage and T-Lymphocyte subpopulations in the Nx model

**Figure 8:**
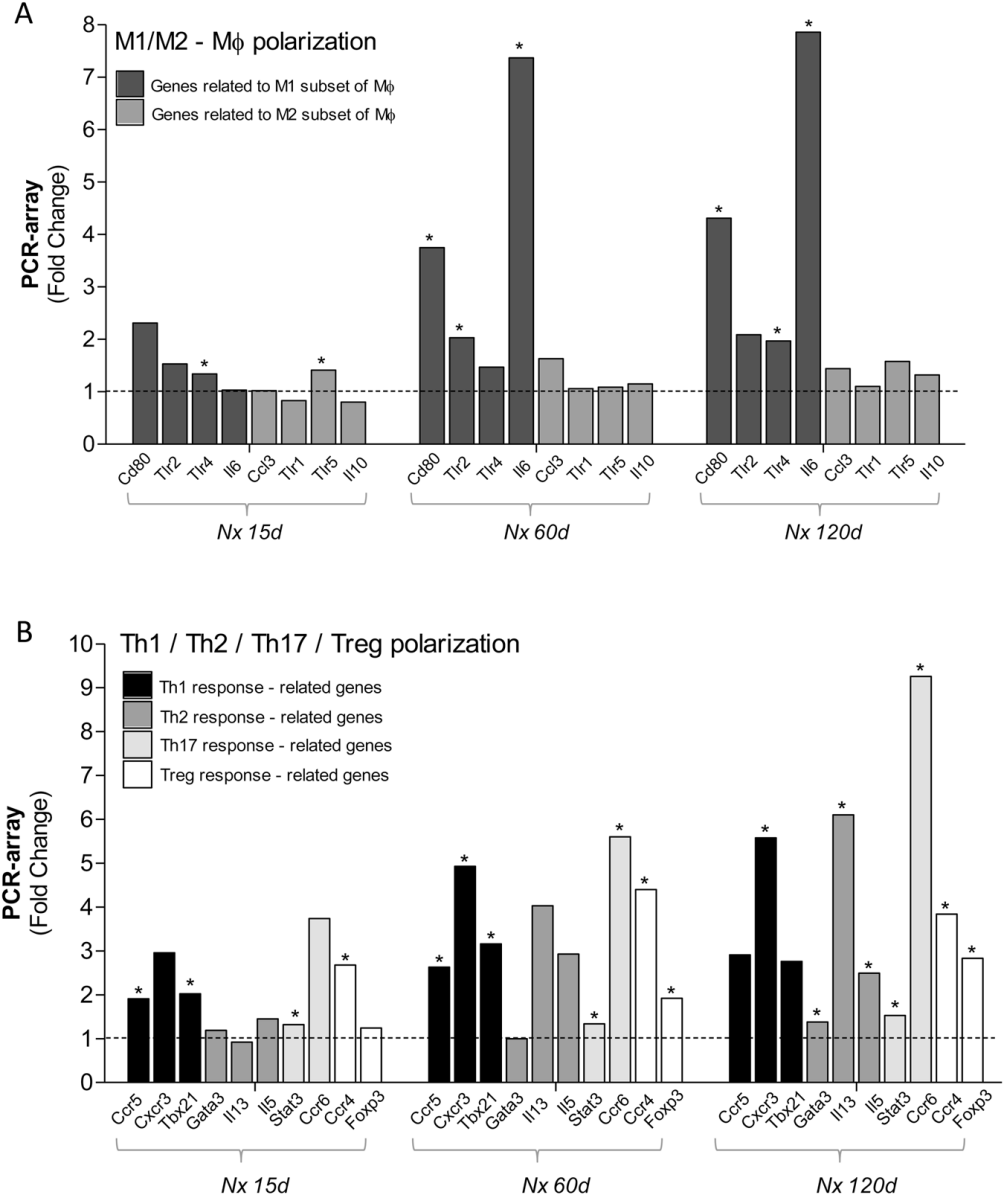
Bar graphs representing the expression of genes related to (**A**) Macrophage (Mϕ) M1 (*Cd80, Tlr2, Tlr4, Il6*) and M2 (*Ccl3, Tlr1, Tlr5, Il10*) subsets and to (**B**) T-Lymphocyte (T-Ly) Th1 (*Ccr5, Cxcr3, Tbx21*), Th2 (*Gata3, Il13, Il5*), Th17 (*Sat3*, *Ccr6*) and Treg (*Ccr4*, *Foxp3*) subsets in the renal parenchyma of Nx rats, using PCR-array analysis. For these experiments, cDNA samples were obtained from nine different animals per group: Sham, Nx 15d, Nx 60d and Nx 120d. These cDNA was than distributed among three pools per group, containing genetic material of three different animals each. For statistical analysis we considered N = 3. Results are presented as mean of fold change, and differences between Nx and Sham were assessed by Student’s t-test (*p < 0.05 vs. Sham), and analyzed with the Rt2 Profiler PCR Array Analysis software, Version 3.5™ (Qiagen).

Of the four genes consistently related to the M1 macrophage phenotype included in this study (*Cd80*, *Tlr2*, *Tlr4*, *Il6*), only *Tlr4* was significantly overexpressed 15 days after Nx. Likewise, only *Tlr5* of the four genes related to the M2 subset of macrophages (*Ccl3*, *Il10*, *Tlr1*, *Tlr5*)^33–35^ was upregulated at this time. Sixty and 120 days after renal ablation 3 of the 4 M1-related genes, and none of the M2-related genes, were significantly overexpressed (Fig. 8A).

As shown in Fig. 8B, the expression of genes related to Th1 (*Ccr5*, *Cxcr3*, *Tbx21*), Th17 (*Stat3*, *Ccr6*) and Treg (*Ccr4*, *Foxp3*)^36–38^ responses, was already elevated 15 days after nephrectomy, increasing further with time. Genes related to the Th2 (*Gata3*, *Il13*, *Il5*)^36–38^ response, were significantly overexpressed only after 120 days of Nx.

## Discussion

As expected, Nx rats exhibited progressive hypertension and albuminuria, as well as creatinine retention and severe glomerular and interstitial injury^1, 5, 6^. Concomitant with these renal lesions, and even preceding them, the presence of inflammatory events such as infiltration by Mϕ and T-Ly was equally conspicuous. Previous studies reported even earlier activation of inflammatory mechanisms in the Nx model^1, 22, 39^, although the possibility that such response merely reflects the acute effect of surgical manipulation cannot be ruled out.

In the present study, we observed evidence of inflammation seven days after Nx (data not shown), corroborating earlier findings^7, 22, 39^. However, we chose to start our analysis fifteen days after renal ablation, when activation of inflammatory mechanisms could no longer be attributed to residual effects of surgical manipulation. At this time, Nx rats exhibited marked activation of innate immunity when compared to Sham. TLRs 4 and 5, known to bind pathogen-associated molecules such as LPS and flagellin^12, 40–44^, along with the *Lbp*, *Cd14*, *Jun* and, not shown previously, *Irf3* and *Irf7* genes, were significantly overexpressed in these animals. The pathogenic role of TLR4 is further suggested by the significant correlation observed between its gene and protein expression and albuminuria, tail-cuff pressure and the percent cortical interstitial area. Our data are consistent with those recently published by Souza and collaborators^24^ showing that TLR4-deficient mice developed less interstitial fibrosis than wild-type controls four weeks after Nx. The contribution of TLR4 may have involved NLRP3 activation in renal tubular cells^24^.

The observation of subsequent time points showed for the first time that, in Nx rats, the TLR2, TLR7 and TLR9 pathways, along with IRF3 and some of its target genes, such as *Cd40* and *Cd86*, start 60 days after renal ablation and remain overexpressed thereafter. These observations reinforce the view that activation of innate immunity in this model does not merely echo an acute reaction to surgery, but, rather, is intrinsically related to CKD progression even as late as 120 days after renal ablation, when rats exhibit severe renal injury that mimic advanced human disease^31^. The potential pathogenic implication of this finding is underlined by the observation that podocyte expression of TLR9 in lupus nephritis associates with the intensity of proteinuria and the progression to CKD^45^. Accordingly, recent studies on *TLR9* polymorphism showed that overexpression of *TLR9* increases the risk of developing CKD in Chinese subjects^46, 47^.

Continued activation of TLR pathways in Nx may have been triggered by danger-associated molecular patterns (DAMPs) such as extranuclear DNA fragments, membrane debris and heat shock proteins, that may have been released by endothelial and/or epithelial cells under mechanical stress (cell stretching by glomerular hypertension and/or hypertrophy) and/or chemical stress (hypoxia, exposure to excess filtered albumin, high glucose concentrations, toxins)^12, 41, 44, 48^. Once activated, the TLR pathways can also stimulate the NF-κB system, triggering additional inflammatory cell events^49, 50^. We have previously shown that NF-κB inhibition strongly attenuated renal injury and inflammation in the Nx and in the adenine overload models^21, 27^, suggesting a key pathogenic role for this pathway. In the present study, we observed progressive upregulation of a growing number of well-known NF-κB target genes, such as *Tnf*, *Il6*, *Il1b* and *Il2*, suggesting sustained NF-κB activation. Accordingly, we showed overexpression of the *Nfkb1* gene at 120 days of Nx. These findings are consistent with our previous observations that renal cortical phosphorylated p65 was increased 60 days after Nx^21^, and with a report of progressive p65 nuclear translocation in Nx rats^50^ reinforcing the notion that the NF-κB system is involved in the renal inflammatory process associated with this model.

Along with activation of TLR pathways and the NF-κB cascade, inflammasomes are known to play a pivotal role in the overall innate immunity response. Assembly of the NLRP3 inflammasome is related to autophagy, apoptosis, extracellular matrix overproduction and synthesis of cytokines and powerful inflammatory mediators such as IL-1β and IL-18^23–26, 51^. Once thought to be present only in leukocytes, NLRP3 inflammasome was recently described in nonimmune cells in the kidney, such as tubular epithelial cells and even podocytes, under stressful conditions^16, 23, 25, 51^. Moreover, NLRP3 inhibition has been shown to be protective against inflammation in experimental murine autoimmune encephalomyelitis, salt-induced hypertension and crystal-induced nephropathy in mice^22, 51–54^. In *Nlrp3* knockout mice subjected to unilateral ureteral obstruction, Vilaysane and coworkers^25^ showed that activation of the NLRP3 pathway is required to the progression of renal fibrosis. Conversely, *Nlrp3* overexpression was shown to worsen inflammation in autoimmune renal disease, and to exacerbate renal injury in experimental diabetic and crystal-related nephropathy^23^.

In consistency with previous observations of the Nx model^22^, we found sustained overexpression of genes directly related to the NLRP3 inflammasome activation (*Casp1*, *Nlrp3* and *Il1b*). Such upregulation was observed as early as 15 days after Nx and persisted throughout the study, supporting a role for this innate immunity pathway in the amplification and propagation of renal inflammation in this model. On the other hand, overexpression of *Nod2*, also related to “sterile inflammation”, was first observed only 60 days after Nx, when glomerulosclerosis was already significant. NOD2 can activate the NF-κB cascade^13, 55, 56^ and has been pointed as one of the critical components of a signal transduction pathway that links renal injury to inflammation in experimental diabetic nephropathy^55^. However, the pathogenic role of NOD2 in CKD is presently unclear. Here we show, for the first time, that activation of this NOD receptor is a late event in Nx, paralleling the development of glomerular injury. Together, these findings suggest that more than one NOD receptor is involved in the development and maintenance of inflammation in the Nx model, although the NLRP3 system seems to be activated in an earlier and more consistent fashion. At more advanced phases (60 days after Nx), the TLR7, TLR9 and NOD2 signaling pathways are set into operation, followed by activation (120 days after Nx), of a TRAM-dependent TLR4 signaling pathway, thus contributing to extend and propagate renal inflammation toward irreversible structural damage.

The role of renal inflammation in the development and progression of hypertension, albuminuria, interstitial and glomerular damage has been extensively shown in both experimental and clinical protocols^1–4^, whereas treatment with anti-inflammatory drugs was reported to exert a renoprotective effect in experimental CKD^6, 14, 58^. In the present study, we were able to pinpoint a host of genes and pathways associated with inflammation that were recruited at different time points along the course of CKD. In particular, we have corroborated previous findings obtained in our laboratory and elsewhere^1, 5–7^, showing that renal cortical infiltration by Mϕ was an early event in Nx rats, gaining intensity steadily with time. More detailed analysis of Mϕ-associated genes suggests that, initially, activation of the M1 and M2 subsets was comparable, a finding compatible with the notion that part of the Mϕ infiltration may have been associated with scavenging and clearing of the renal parenchyma^33–35^. At subsequent time points, however, a clear predominance of genes encoding M1 putative markers was observed. “Classically” activated M1 macrophages are known to release reactive oxygen species and inflammatory cytokines including TNF, IL-1 and IL-6. M1 polarization has been characterized in a variety of CKD models, such as ureteral obstruction, doxorubicin-induced nephropathy and diabetic nephropathy^35^. Our findings suggest that infiltrating Mϕ may be progressively oriented toward a tissue damaging phenotype in the Nx model as well.

The present observations reinforce previous evidence from our laboratory and elsewhere^28, 29^ that adaptive immunity is central to the pathogenesis of CKD: besides the significant correlation observed between CD3 expression and the GS index, progressive renal T-Ly infiltration was seen since 15 days after Nx, along with overexpression of a number of genes knowingly related to the Th1, Th17 and Treg responses. In addition, we showed that the expression of these genes, along with that of several other genes linked to innate immunity or inflammation, increases steadily with time. As a whole, these observations help to explain why therapeutic agents and schemes shown to largely prevent CKD when given early in the course of the disease lose efficacy when treatment is initiated at late phases^29, 30, 59^, after an overwhelming number of genes and pathways has been activated.

Although the complex net of pathogenic pathways involved in CKD may look discouraging, this study revealed that several genes linked to innate and adaptive immunity, such as *Cd14*, *Itgam*, *Ccr4*, *Ccr5*, *Ccr6* and *Ccr8*, among many others, are activated and remain so until advanced phases of the Nx model. It is conceivable that one or more of these late activated molecules and/or pathways be responsible for the refractoriness of advanced CKD to treatments aimed at detaining it, and may serve as therapeutic targets in the future.

**Figure 9:**
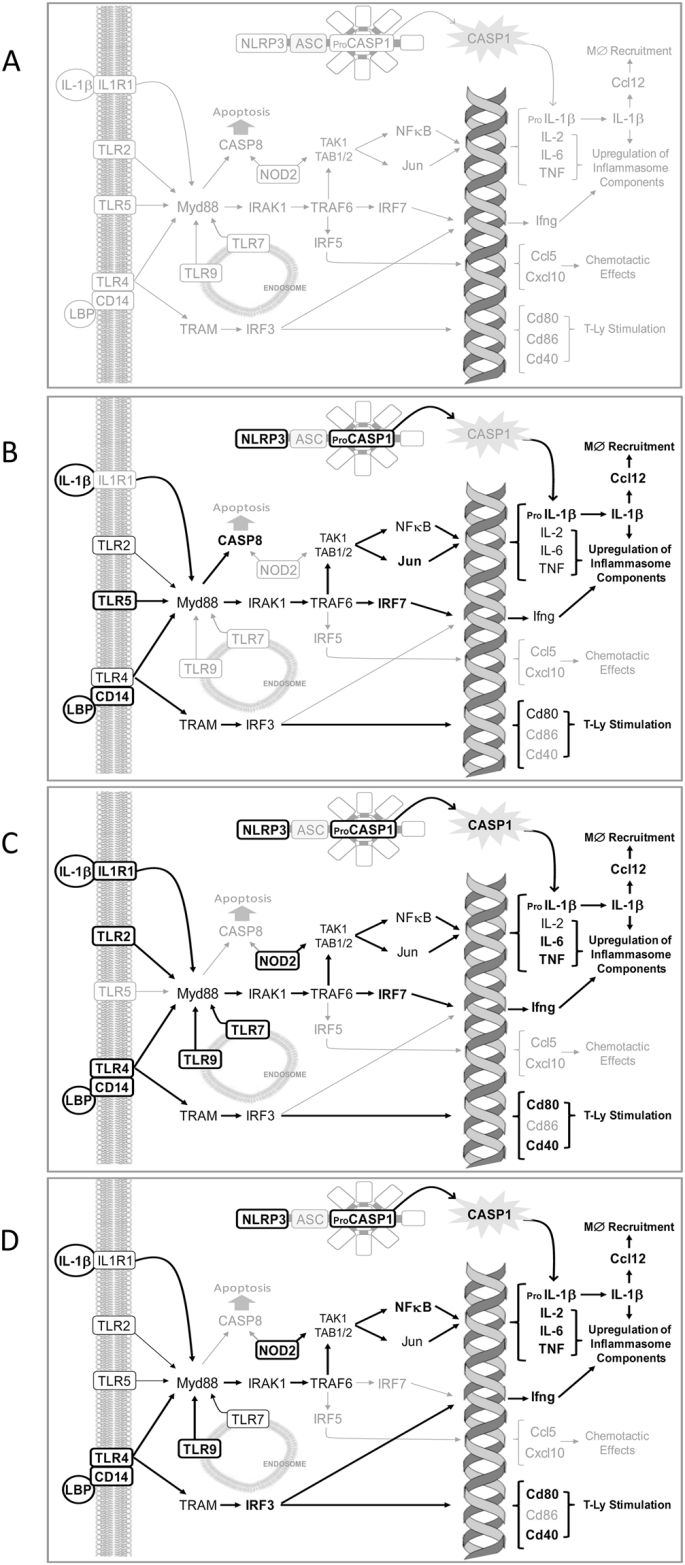
Illustration of a proposed signaling pathway of innate and adaptive immune activation in the Nx model (**A**). Illustrations of proposed maps of activation of TLRs, NF-κB and inflammasomes pathways at each point of the study are represented respectively in (**B**) Nx 15d, (**C**) Nx 60d and (**D**) Nx 120 days. The elements represented in black are overexpressed and those represented in bold are statistically upregulated.

Based on the present results, and in the light of the current literature^17, 40, 41, 57, 60–62^, we propose a speculative map of innate immunity activation along the course of CKD in the Nx model (Fig. 9), with emphasis on the TLR pathways, the NF-κB system and the inflammasomes. The MyD88 adaptor protein may exert an important role in this chain of events, since it acts as an IL-1R intracellular signaling adaptor^63^. These concepts are consistent with our previous observation that MyD88 knockout mice submitted to adenine excess showed reduced gene and protein expression of IL-1β, and were protected against tubulointerstitial nephritis, compared to WT animals^17^. They are also in line with the observed overexpression of *the Irf7* gene, which was sustained throughout the present study.

Despite of its observational nature, and although it was heavily based on gene analysis, the present study brings novel information regarding the immune pathways set in motion in the Nx model, and the timing of their activation. As a whole, these findings indicate that both innate and adaptive immune responses are triggered since early phases of the Nx model, remaining activated until very advanced stages, underlining the intricate nature of the process leading to CKD, and illustrating the difficulty in finding adequate therapeutic targets among this complex and often redundant chain of events.

## Methods

### Animals and Experimental Model

Eighty adult male Munich-Wistar rats weighing 220–250 g were obtained from a local facility at the University of São Paulo. All experimental procedures were specifically approved by the local Research Ethics Committee (CAPPesq, process No. 272/12), and developed in strict conformity with our institutional guidelines and with international standards for manipulation and care of laboratory animals. Rats were monitored daily for assessment of body weight gain and general condition. Animals with limited mobility, reduced food and fluid intake and/or severe body weight stunting were excluded from the study.

Five-sixths renal ablation (Nx) was performed in a single-step procedure. Sixty rats were anesthetized with an intramuscular injection of ketamine (50 mg/kg) and xylazine (10 mg/kg) and subjected to a ventral laparotomy. The right kidney was removed and two or three branches of left renal artery were ligated, resulting in the infarction of two-thirds of the left kidney. After full recovery, all animals received enrofloxacin, had free access to tap water and rodent chow containing 0.5 Na and 22% protein (Nuvital Labs, Curitiba, Brazil), and were kept at 23 ± 1 °C and 60 ± 5% relative air humidity, under an artificial 12–12 hour light/dark cycle. Nx animals were followed for 15 days (N = 20), 60 days (N = 20) or 120 days (N = 20) after surgery. At each of these time points, tail-cuff pressure (TCP, mmHg) was measured with an automated optoelectronic device (Visitech Systems, Apex, NC) and 24-h urinary albumin excretion (U_alb_V, mg/day) was determined. Rats were then anesthetized with ketamine, (50 mg/kg) and xylazine (10 mg/kg) IM, and blood was collected from the abdominal aorta for assessment of serum creatinine concentration (S_Cr_, mg/dL) using a commercially available kit (Labtest Diagnostica, São Paulo, Brazil). The kidneys of ten rats of each group were retrogradely perfusion-fixed through the abdominal aorta with Dubosq-Brazil solution after a brief washout with saline to remove blood from the renal vessels. After weighing, two midcoronal renal slices were postfixed in buffered 4% formaldehyde and embedded in paraffin through conventional sequential techniques. The remaining 10 rats of each group had their kidneys excised and rapidly frozen for subsequent protein and RNA extraction. Twenty sham-operated rats (Sham) were used as controls. These rats were anesthetized and underwent ventral laparotomy and renal manipulation without any reduction of renal mass. Preliminary experiments showed that renal function and innate immunity expression were stable in these animals throughout the study (data not shown), so we followed Sham animals for 120 days after surgery, and used this group as the control for all analysis.

### Histological and Immunohistochemical Analysis

Histomorphometric and immunohistochemical analyses of the renal tissue were performed in 4-mm-thick sections. The extent of GS was estimated, in sections stained with periodic acid Schiff (PAS), by assigning a score to estimate the sclerosing fraction of the tuft area of each glomerulus (at least 120 per rat were examined), and deriving a GS index (GSI) for each rat, as described previously^64^. The percentage of renal cortical interstitial area (INT) was estimated in trichrome-stained sections by a point-counting technique^65^. Immunohistochemical analysis (IHC) was performed to Mϕ, and T-Ly, cells/mm^2^ in renal cortex, as described previously^61^. Mϕ and T-Ly were detected by monoclonal mouse anti-ED1 (Serotec #MCA341R) and anti-CD3 (Dako #M7254) antibodies, respectively. For TLR4 assessment, sections were deparaffinized and rehydrated by conventional techniques, then heated in citrate buffer for antigen retrieval and pretreated with 30% hydrogen peroxide in methanol for endogenous peroxidase blockade. Sections were preincubated with protein-block serum-free solution (Dako, #X0909) to avoid nonspecific binding, then incubated overnight at 4 °C with a polyclonal rabbit anti TLR4 antibody (Santa Cruz #SC30002), diluted in bovine serum albumin at 1%. The EnVision Labelled Polymer for peroxydase (Dako, Denmark) was used before development with DAB substrate. The percentage of TLR4-positive renal tubules and the intensity of interstitial infiltration (cells/mm^2^) by TLR4-positive cells were estimated in at least 30 different microscopic fields per rat, under a 400x magnification.

### Western Blot and ELISA

For Western Blot (WB) analysis, frozen samples of renal cortex weighing 50–100 mg were lysed in RIPA buffer. An amount of 100 μg of total protein was then run on a 10% SDS-PAGE gel, as described elsewhere^27^. Proteins were transferred onto a nitrocellulose membrane (Amersham Biosciences, Little Chalfont, UK) and the expression of the CASP1 protein was evaluated through the incubation of such membranes with a primary polyclonal rabbit anti-CASP1, antibody (#SC56036, Santa Cruz), using manufacturer recommended dilutions, followed by a peroxidase-conjugated antibody (Sigma). Bands were detected using an enhanced chemoluminescence system and analyzed with the Uvitec Cambridge® gel documentation device. Finally, membranes were stripped and probed with a primary anti-β-Actin antibody (Sigma) to confirm and estimate the loading and transfer proceedings. Further protein lysates obtained from frozen samples of renal cortex were used for ELISA analysis of the content of interleukin 1β (protein IL-1β) with a commercially available kit (#SRLB00, R&D Systems). This result was expressed as pg/ng of protein.

### Gene Analysis

Total RNA was obtained from frozen samples of renal cortex with a commercially available column-based nucleic acid purification kit (RNeasy, Qiagen® #74106), following strictly the instructions of the manufacturer. Kidney samples were placed in reinforced polypropylene tubes containing ceramic beads (Omni Bead Ruptor tubes #19-628) and the appropriate lysis buffer. Samples were stirred in the Omni Bead Ruptor 24® homogenizer, then subjected to a multi-step protocol of washing and centrifugation. After evaluation of RNA purity and final concentration, cDNA was synthesized through reverse transcription (RT) using the Rt² Easy First Strand kit (Qiagen® #330401).

PCR-array analysis was employed to screen for alterations in the expressions of genes related to innate and adaptive immunity. For this, cDNA obtained from 9 animals of each experimental group was distributed among three pools, derived from 3 animals each. The cDNAs were mixed with the appropriate PCR master mix buffer (Rt² Syber Green ROX qPCR Primer Assay QiagenN® #330523) and analyzed on specific array plates (Rat Innate & Adaptive Immune Responses Rt² Profiler™ PCR, Qiagen®, #330231-PARN052Zc).

We further analyzed the expression of selected genes involved in the innate immune response by individual qPCR (10 animals/group). Commercially available pre-validated primers (Rt² qPCR Primer Assay Qiagen #330001 series) were employed to assess the expression of *Tlr4* (#PPR45931B; NM_0191178), *Nlrp3* (#PPR56639A; NM_001191642), *Casp1* (#PPR06427A; NM_012762), *Il1b* (#PPR06480B; NM_031512) and the housekeeping gene *Actb* (#PPR06570C; NM_031144).

Both PCR-array and qPCR analysis were performed using a ViiA™7 Real-Time PCR System (Life Technologies do Brasil, São Paulo) and the results assessed with the 12 K Flex QuantStudio^TM^ software (Applied Biosystems, Foster City, CA). The expression of all genes of interest is presented as fold change relative to Sham, as described earlier^17^. Further pathway-focused gene analyses were performed based on the Kyoto Encyclopedia of Genes and Genomes (Kegg) Pathway online database^57^ and the “Enrichr” gene enrichment analysis online tool^57, 60–62^.

### Statistical Analysis

Results are presented as Mean ± 1 SE. Differences among groups were analyzed by one-way ANOVA with Newman-Keuls post tests, while correlations were established according to the Pearson’s correlation coefficient, both using Graph-Pad Prism^TM^ (version 5.0). PCR-array results (vs. Sham) were analyzed by Student’s t-test using the 12 K Flex QuantStudio™ software (Applied Biosystems, Foster City, CA). Differences among groups and correlations among different parameters were considered statistically significant when p < 0.05.

## Electronic supplementary material

Supplementary Information
