# Supplementary material for: Innate And Adaptive Immunity are Progressively Activated in Parallel with Renal Injury in the 5/6 Renal Ablation Model
Source: Sci Rep. 2017 Jun 9;7:3192. doi: 10.1038/s41598-017-02915-6 (PMC5466605; doi:10.1038/s41598-017-02915-6)
Supplement: Supplementary file 1 — Supplementary Information [file 41598_2017_2915_MOESM1_ESM.doc]

INNATE AND ADAPTIVE IMMUNITY ARE PROGRESSIVELY ACTIVATED IN PARALLEL WITH RENAL INJURY IN THE 5/6 RENAL ABLATION MODEL

Camilla Fanelli, PhD, Simone CA Arias, PhD, Flavia G Machado, PhD, Jessica K Okuma, Denise MAC Malheiros, MD, PhD, Hatylas Azevedo, Carlos A Moreira-Filho, MD, PhD, Niels OS Camara, MD, PhD, Clarice K Fujihara, PhD, Roberto Zatz, MD, PhD

Faculty of Medicine, University of São Paulo, São Paulo, Brazil

**Running headline:** Innate and adaptive immunity in the remnant model of CKD

**Corresponding author:**

Roberto Zatz

Av. Dr. Arnaldo, 455, 3-s/3342

01246-903, São Paulo, SP, Brazil,

Phone: +55-11-3061-7260 Fax: +55-11-3061-7261

E-mail: [roberto.zatz@gmail.com](mailto:roberto.zatz@gmail.com)

**Supplementary Information**

Full-length Pro-CASP1, CASP1 (A) and constitutive β-Actin (B) representative Western Blots of groups Sham, Nx 7d (not included in the study; see Discussion, second paragraph), Nx 15d, Nx 60d and Nx 120d.
